# Supplementary material for: “Pulsed Hypoxia” Gradually Reprograms Breast Cancer Fibroblasts into Pro-Tumorigenic Cells via Mesenchymal–Epithelial Transition
Source: Int J Mol Sci. 2023 Jan 27;24(3):2494. doi: 10.3390/ijms24032494 (PMC9916667; doi:10.3390/ijms24032494)
Supplement: Supplementary file 1 [file ijms-24-02494-s001.zip › ijms-2129858-supplementary.pdf]

Supplementary Materials:

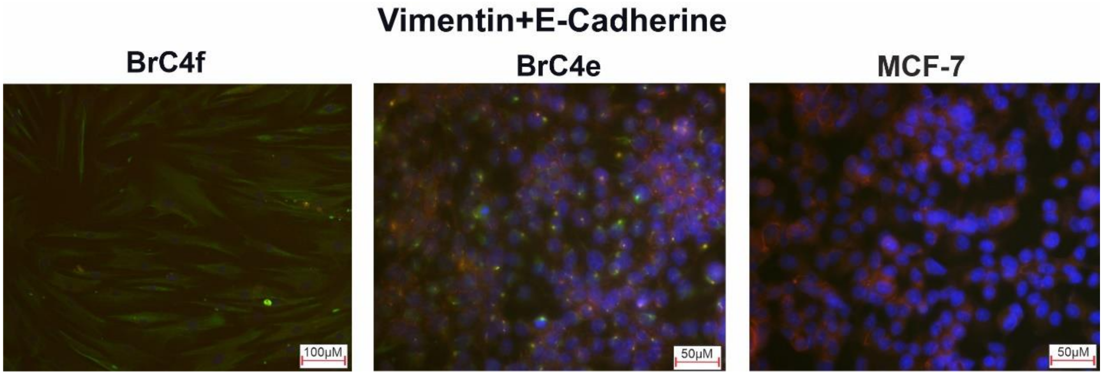

Figure S1. Detection of vimentin and E-cadherin after transformation of fibroblast cells to epithelial cells.

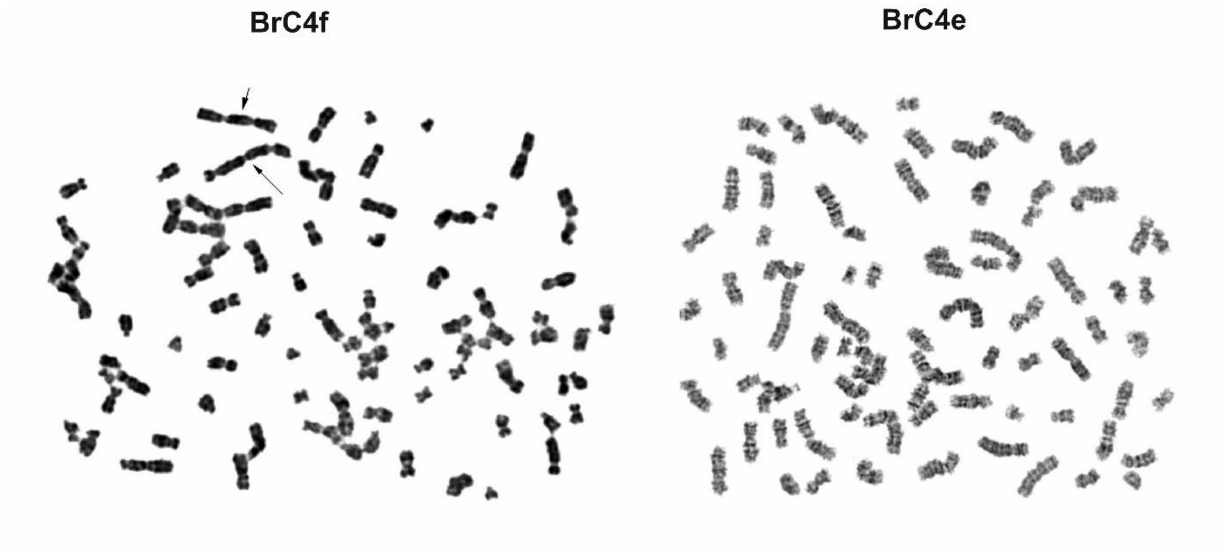

Figure S2. Karyotype analysis of GTG-stained chromosomes.

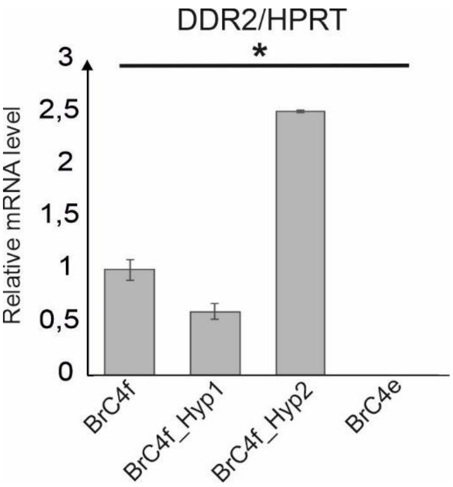

Figure S3. Alteration of mRNA expression level DDR2 gene by RT-PCR. The expression of specific mRNAs was normalized to the expression level of Hypoxanthine Phosphoribosyl transferase (HPRT) mRNA. Statistical analysis included the results of two independent experiments (mean ± SD). * The difference between the experimental group and the control (BrC4f) was statistically significant at $p < 0.05$.

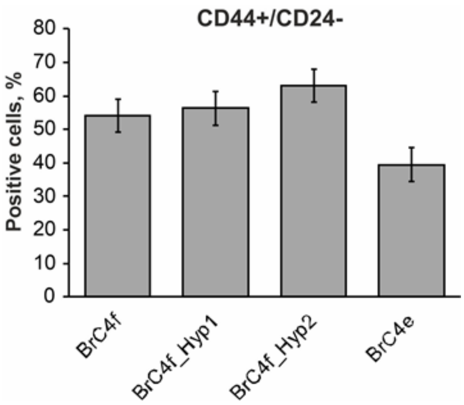

Figure S4. CD44$^+$/CD24$^-$ population in the established cell lines. Data of flow cytofluoromety analysis of immunostained cells.
